# Supplementary material for: Probing the E1o-E2o and E1a-E2o Interactions in Binary Subcomplexes of the Human 2-Oxoglutarate Dehydrogenase and 2-Oxoadipate Dehydrogenase Complexes by Chemical Cross-Linking Mass Spectrometry and Molecular Dynamics Simulation
Source: Int J Mol Sci. 2023 Feb 25;24(5):4555. doi: 10.3390/ijms24054555 (PMC10003691; doi:10.3390/ijms24054555)
Supplement: Supplementary file 1 [file ijms-24-04555-s001.zip › ijms-2139730-supplementary.pdf]

Supporting Information

Probing the E1o-E2o and E1a-E2o Interactions in Binary Subcomplexes of the Human 2-Oxoglutarate Dehydrogenase and 2-Oxoadipate Dehydrogenase Complexes by Chemical Cross-linking Mass Spectrometry and Molecular Dynamics Simulation

Oliver Ozohanics[2], Xu Zhang[1], Natalia S. Nemeria[1], Attila Ambrus[2*], and Frank Jordan[1*]

[1] Department of Chemistry, Rutgers University, Newark, New Jersey 07102, USA

[2] Department of Biochemistry, Institute of Biochemistry and Molecular Biology, Semmelweis University, Budapest, 1094, Hungary

*Correspondence: Frank Jordan E-mail: frjordan@newark.rutgers.edu

Attila Ambrus E-mail: ambrus.attila@med.semmelweis-univ.hu

Table S1. The inter-component cross-links identified by CL-MS in the hE1o-hE2o and hE1a-hE2o binary sub-complexes 2,3

Figure S1. Multiple sequence alignment of the hE1o protein.. 4

Figure S2. The comparison of the Q1 *vs.* Q2 analysis of the native contacts for the hE1o-hE2o and hE1a-hE2o sub-complexes 5

Figure S3. The RMSF plots for the hE1o and hE1a subunits.. 6

Figure S4: The RMSD plots for the hE2o core domain... 7

Table S1. The inter-component cross-links identified by CL-MS in the hE1o-hE2o and hE1a-hE2o binary sub-complexes. The expected distance constraints compared to those calculated from the best oriented docked structures. Cross-links found within the expected distance are colored in *green*.

Residue number in hE2o	Residue number in hE1o	Calculated distance in Best pose 1*	Calculated distance in Best pose 2	Residue number in hE2o	Residue number in hE1a	Calculated distance in Best pose 1	Calculated distance in Best pose 2*
24	728	46.3	45.2	24	886	35.9	29.4
66	959	55.2	56.2		628	32.5	29.1
78	308	31.6	30.4	43	916	42.5	31.2
	361	27.7	19.2	66	916	29.2	17.8
85	30	14.8	15.0	78	300	22.3	25.2
	488	20.3	19.4		568	26.1	31.6
	499	5.5	4.2	85	628	11.6	14.6
	534	39.9	37.4		827	41.9	37.4
87	30	13.5	13.3	87	143	10.2	18.0
	903	71.9	71.3		388	6.3	6.3
	907	75.4	74.9		553	33.4	32.3
98	494	37.1	37.0		886	39.1	38.6
150	82	18.8	15.9	98	300	17.7	14.0
	308	58.4	58.2		886	63.1	60.2
159	231	61.3	57.4	159	481	15.0	25.7
	657	30.7	35.2	200	69	32.5	22.8
172	236	64.3	64.7	206	481	18.2	26.5
	347	44.2	47.2	286	110	44.3	7.9
200	959	80.4	78.7		148	18.7	11.0
205	521	50.1	47.3		155	22.0	16.3
210	82	6.4	8.7		244	23.8	19.2
	144	14.8	14.8		300	27.6	25.5
	494	53.4	52.0		562	41.8	31.8
240	82	36.6	40.1		628	28.0	25.1
	231	64.0	65.8	289	562	41.4	33.6
	347	42.2	44.6		656	20.9	19.6
286	34	16.6	13.9	342	110	82.0	34.7
289	82	19.6	13.5	371	72	31.9	20.0
	959	60.0	59.5		143	38.8	34.8
342	82	35.2	38.4	373	72	32.2	24.6
371	308	75.9	75.5		110	64.4	20.0
					143	43.8	39.5
					148	35.5	33.5

	244	31.7	23.9
	300	48.8	45.1
	562	58.4	44.8
	628	52.7	52.1

* indicates the orientation applied in the starting structure for MD simulation.

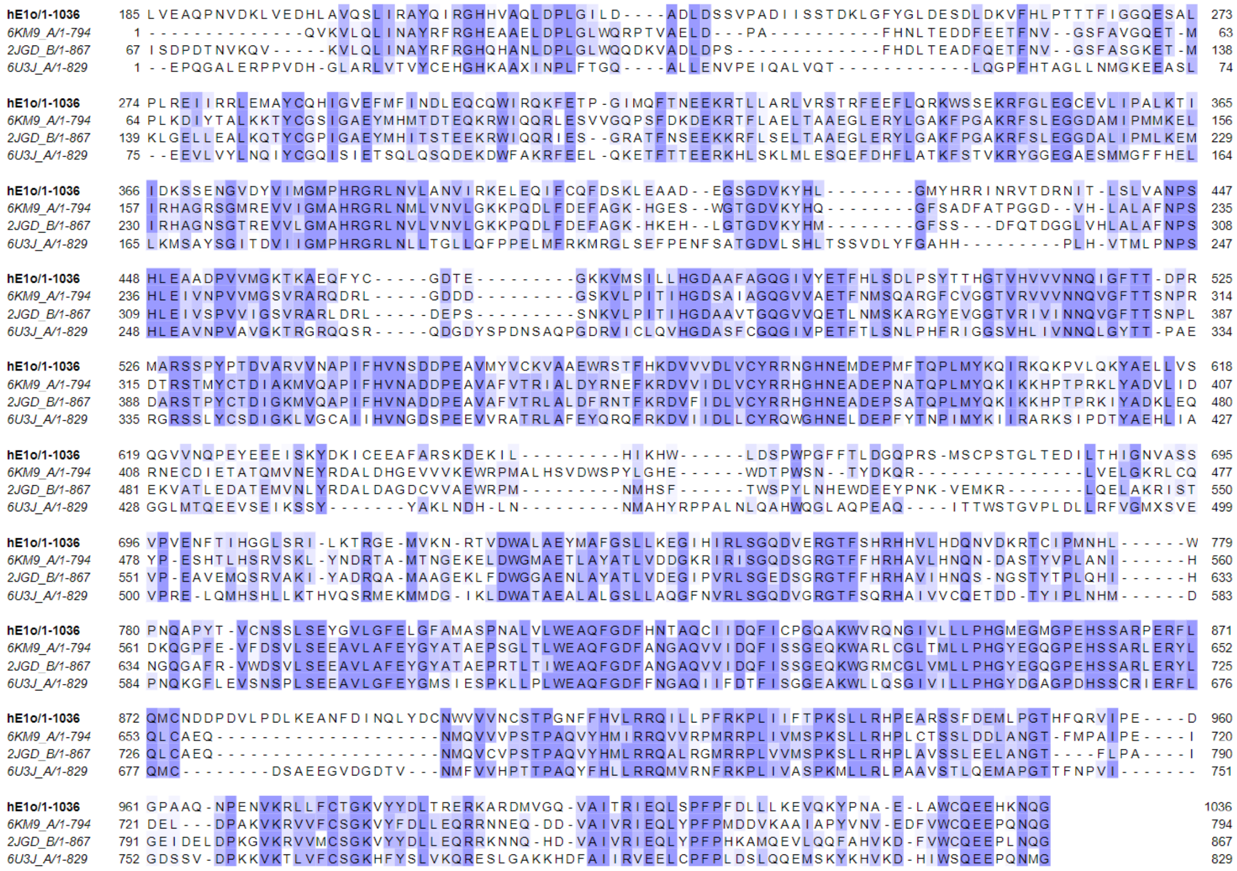

Figure S1. Multiple sequence alignment of the hE1o proteins. The image depicts randomly selected high-scoring hE1o proteins for illustration purpose only and residues are colored by conservation.

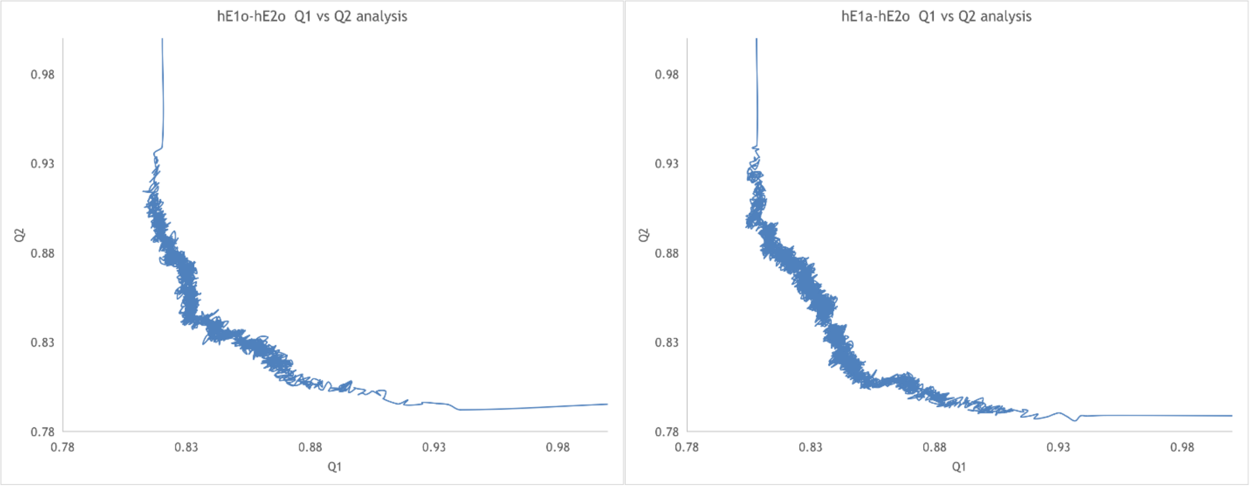

Figure S2. The comparison of the Q1 *vs.* Q2 analysis of the native contacts for the hE1o-hE2o and hE1a-hE2o sub-complexes. Q1 measures the contacts present at the beginning of the simulation, while Q2 measures the contacts of the conformation reached after 50 ns simulation.

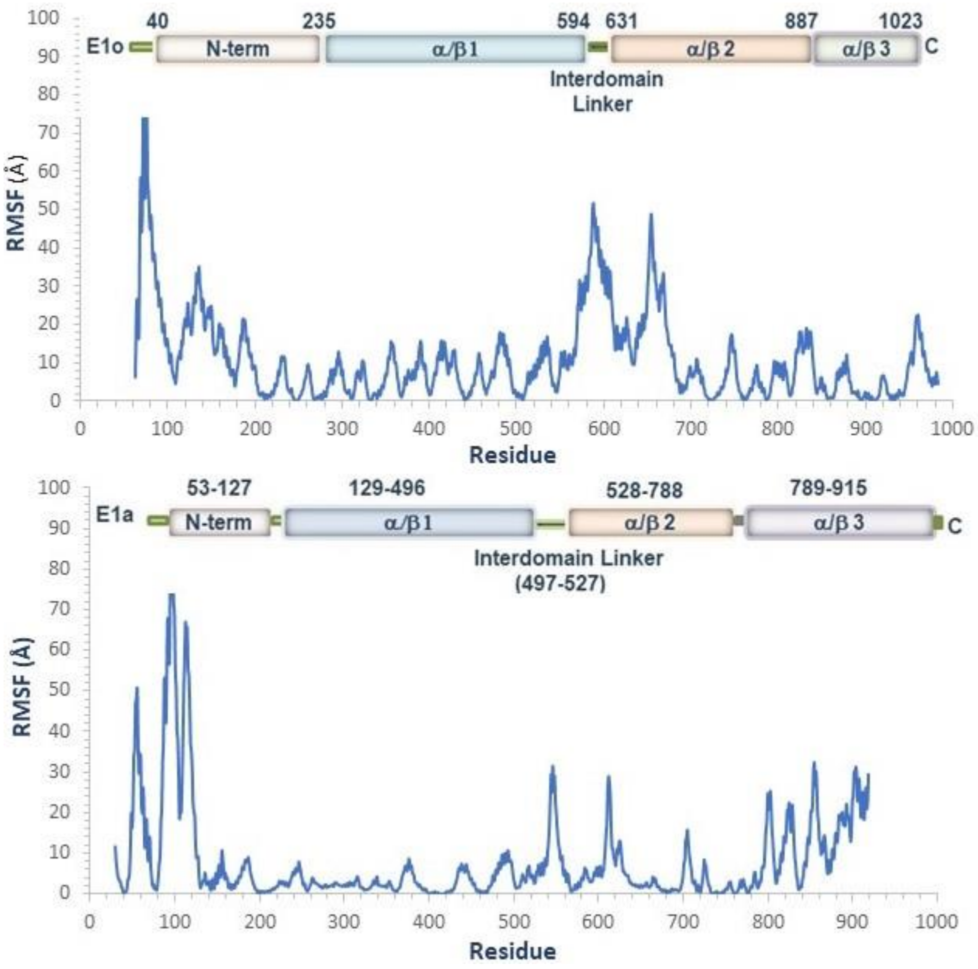

Figure S3. The RMSF plots for the hE1o and hE1a subunits. The schematic representations of the domain structures is based on the reported X-ray structure of hE1a and cryo-EM structure of hE1o. Lower flexibility in the interdomain linker region and a floppier C-terminus are observed in hE1a relative to hE1o.

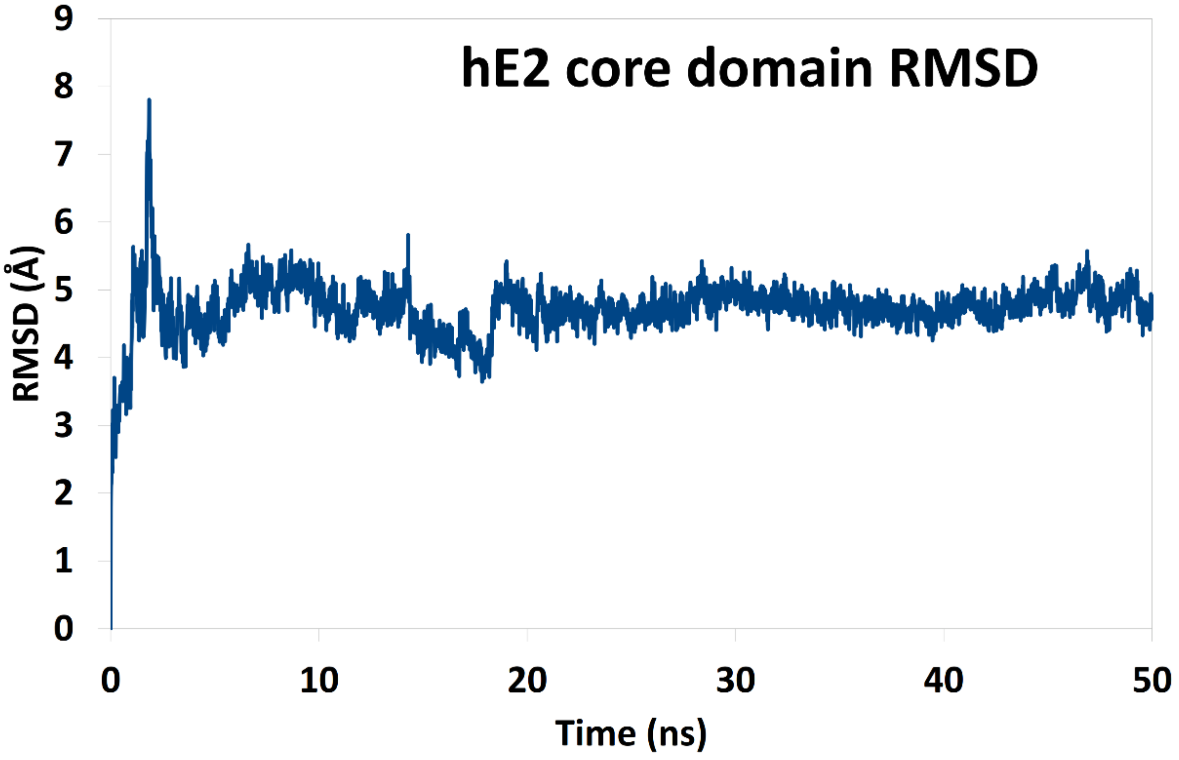

Figure S4. RMSD plot for the hE2o core domain.
